# Comparative Analysis of Corneal Morphological and Optical Parameters in Predicting DSAEK Surgery Outcome

**DOI:** 10.3390/medicina61061022

**Published:** 2025-05-31

**Authors:** Antonela Geber, Sanja Masnec, Miro Kalauz, Iva Bešlić, Ivan Škegro, Dina Lešin Gaćina, Sonja Jandroković, Ana Meter, Tomislav Kuzman

**Affiliations:** 1 Department of Ophthalmology, University Hospital Centre Zagreb, School of Medicine, University of Zagreb, 10000 Zagreb, Croatia; antonelageber@gmail.com (A.G.); miro.kalauz@gmail.com (M.K.); iva.lukac@gmail.com (I.B.); ivanskegro@yahoo.com (I.Š.); dina.lesin@yahoo.com (D.L.G.); sonja.jandrokovic@gmail.com (S.J.); tomislav.kuzman@kbc-zagreb.hr (T.K.); 2 Department of Ophthalmology, University Hospital Dubrava, 10000 Zagreb, Croatia; imeter@yahoo.com

**Keywords:** cornea, corneal transplantation, Descemet stripping automated endothelial keratoplasty, corneal imaging

## Abstract

*Background and Objectives*: Descemet stripping automated endothelial keratoplasty (DSAEK) is a widely used surgical technique for treating corneal endothelial dysfunctions such as Fuchs endothelial corneal dystrophy (FECD) and pseudophakic bullous keratopathy (PBK). This study aimed to investigate the association between postoperative visual acuity and various corneal morphological and optical parameters, including corneal densitometry (CD) and higher-order aberrations (HOAs), measured using the Pentacam (OCULUS Optikgeräte GmbH, Wetzlar, Germany), as well as graft thickness, which was assessed by anterior segment optic coherence tomography (AS-OCT), (Optovue Inc., Fremont, CA, USA), and corneal thickness, assessed by both AS-OCT and Pentacam. *Materials and Methods*: This prospective, observational cohort study included 17 eyes from 13 patients who underwent DSAEK. Data on preoperative visual acuity were collected, while postoperative parameters were assessed during follow-up visits. Corneal measurements included the total corneal and corneal graft thickness, corneal densitometry in 20 defined subregions, and corneal higher-order aberrations. Associations between these parameters and postoperative visual acuity were evaluated using nonparametric statistical tests. *Results*: The postoperative visual acuity improved significantly (*p* < 0.001). Strong correlations were found between poorer visual acuity and higher CD values. The strongest correlations with visual acuity were found for CD 2–6 mm total (Rho = 0.795; *p* < 0.001), CD central 2–6 mm (Rho = 0.791; *p* < 0.001), and CD central 0–2 mm (Rho = 0.730; *p* < 0.001). Significant associations were also observed with anterior and posterior HOAs (Rho = 0.624, *p* = 0.01; and Rho = 0.556, *p* = 0.02, respectively). No correlation was found between visual outcomes and graft thickness measured by AS-OCT (Rho = 0.051; *p* = 0.85), nor with total corneal thickness measured by AS-OCT (Rho = −0.227; *p* = 0.38) or Pentacam (Rho = −0.369; *p* = 0.14). *Conclusions*: This study demonstrates that CD and HOAs are more strongly associated with postoperative visual acuity after DSAEK than traditionally monitored parameters such as graft or corneal thickness. The results highlight the value of detailed corneal imaging and support the use of advanced optical diagnostics in postoperative evaluation.

## 1. Introduction

The corneal endothelium, the innermost layer of the cornea, is composed of a single layer of hexagonal cells and plays a vital role in maintaining corneal transparency and function by regulating the fluid balance within the stromal tissue [1].

Endothelial keratoplasty (EK) is required when corneal transparency is reduced owing to endothelial dysfunction, such as in Fuchs endothelial corneal dystrophy (FECD) and bullous keratopathy (BK) [2]. EK has largely replaced penetrating keratoplasty (PKP) for treating endothelial disorders.

Descemet stripping automated endothelial keratoplasty (DSAEK) is a technique that selectively removes the damaged endothelium and replaces it with donor tissue, including the endothelium, Descemet membrane, and part of the donor stroma [3].

FECD is one of the most common indications for corneal transplantation in Western countries. Due to its strong association with aging, it often co-occurs with cataracts, and many patients undergo a triple DSAEK procedure—a combined surgery involving phacoemulsification, intraocular lens (IOL) implantation, and DSAEK [4].

Compared with PKP, the DSAEK technique offers several advantages, including better postoperative visual acuity, improved contrast sensitivity, and reduced surgery-induced astigmatism and higher-order aberrations (HOAs) [5].

In an effort to improve the anatomical precision and visual outcomes of endothelial keratoplasty, several modified techniques have been developed over time, focusing on reducing the graft thickness and excluding certain structural components of the donor cornea. A newer technique known as Descemet’s membrane endothelial keratoplasty (DMEK) was introduced in 2002 by Melles and colleagues. This approach only utilizes the donor’s Descemet membrane and endothelium, making it a more anatomically precise procedure compared to conventional DSAEK [6]. Although DMEK offers faster visual recovery, better acuity, and lower rejection rates compared to DSAEK, its adoption is limited due to technical challenges and higher complication rates [7,8,9]. Moreover, despite promising outcomes, many patients still fail to reach optimal vision, suggesting that additional factors influence the final visual results [8,10]. There is also the Ultrathin DSAEK (UT-DSAEK) procedure, which is a modified version of the standard DSAEK technique involving a double microkeratome pass to produce thinner grafts with the goal of improving visual outcomes. Previously, UT-DSAEK referred to corneal grafts that were thinner than 130 μm, whereas today, it refers to grafts that are thinner than 100 μm. These definitions are based on a consensus among surgeons and are not strictly standardized, as confirmed by Chamberlain’s study, in which 56% of experienced surgeons define graft thickness below 100 μm as ultrathin, and above 100 μm as the conventional DSAEK method [11]. The current literature continues to explore the extent to which the surgical technique, the donor tissue preparation, graft characteristics such as graft thickness, and recipient-specific factors including corneal thickness and other corneal parameters influence the clinical outcomes following DSAEK [12,13,14,15,16,17,18].

The Pentacam is an anterior segment imaging device based on the rotating Scheimpflug camera principle. It captures high-resolution, cross-sectional images of the cornea, enabling detailed visualization of the corneal structure. In addition to mapping the corneal thickness and curvature, the device performs corneal densitometry (CD) by quantifying the light backscatter, allowing for the assessment of tissue reflectivity across various corneal layers and concentric zones. It also provides a Zernike analysis for evaluating corneal HOAs [19].

The aim of this study is to investigate the association between corneal parameters, measured using Pentacam and anterior segment optic coherence tomography (AS-OCT), and postoperative visual acuity in patients who underwent a DSAEK procedure.

The study hypothesizes that both morphological parameters measured by Pentacam, such as CD, and optical parameters, such as HOAs, show a stronger correlation with postoperative visual acuity following DSAEK surgery than the commonly measured parameters of corneal thickness, measured by both Pentacam and AS-OCT, and graft thickness, measured by Pentacam.

## 2. Materials and Methods

This prospective, observational cohort study included 24 eyes from 20 patients who underwent a DSAEK procedure at the Department of Ophthalmology, University Hospital Center Zagreb, between June 2022 and November 2024. All patients signed informed consent for participation in the study, which was approved by the Ethics Committee of the University Hospital Center Zagreb (protocol code 8.1-25/11-2 number 02/013 AG; date of approval: 3 February 2025).

Donor corneal grafts were precut and preserved using conventional techniques at the Eye bank of University Hospital Centre Zagreb. The corneas were stored either in hypothermic storage or in tissue culture media. Microbiological testing was performed during storage to ensure safety.

Preparation of the corneas for DSAEK was carried out by specially trained staff using an automated microkeratome (Gebauer SLc Original, Neuhausen, Germany). Surgeries were performed under general anesthesia or augmented local anesthesia by two experienced anterior segment surgeons (T.K., M.K.).

In the triple DSAEK procedure, following phacoemulsification and IOL implantation, Miostat (Carbachol 0.01%, Alcon, Switzerland SA, Risch, Switzerland) was injected into the anterior chamber to induce miosis. Under continuous irrigation, a peripheral iridotomy was performed at the 6 o’clock position using vertical vitreoretinal scissors to prevent potential postoperative pupillary block.

An 8.0 mm diameter descemetorhexis was performed using a reverse Sinskey hook (Moria Surgical, Antony, France). The central Descemet membrane was removed using endothelial 23-gauge grasping forceps (Moria Surgical, Antony, France). An 8.0 mm trephine was then used to trephinate the donor graft. The donor graft was inserted into the anterior chamber using a Busin glide (Moria Surgical, Antony, France) through the main incision. An air bubble was injected into the anterior chamber to press the graft against the stroma. Finally, reconstituted cefuroxime (1 mg/0.1 mL) was injected into the anterior chamber.

None of the patients had early postoperative complications such as graft dislocation and interface fluid; therefore, none of the patients underwent postoperative rebubbling.

While preoperative data and donor information were collected retrospectively from medical records and the Eye bank database, postoperative data were obtained during scheduled follow-up visits, specifically arranged for the purpose of this study.

Seven eyes were subsequently excluded from the study based on predefined criteria, resulting in a final analysis of seventeen eyes. The exclusion criteria included a history of previous corneal transplantation, the presence of other corneal pathologies, severe retinal or optic nerve diseases, as well as systemic conditions such as uncontrolled diabetes mellitus or autoimmune disorders that could affect corneal healing. Additionally, patients with graft failure within the first 3 months or those requiring additional surgical interventions during the follow-up period were also excluded from the analysis.

Collected data included demographic information about patients, indications for surgery, and the type of surgical procedure. Indications for surgery included Fuchs endothelial corneal dystrophy (FECD) and pseudophakic bullous keratopathy (PBK). The type of surgery was also recorded, distinguishing between isolated DSAEK and the triple procedure, which combines phacoemulsification and IOL implantation with DSAEK.

Furthermore, data on preoperative best-corrected visual acuity (BCVA), as recorded upon hospital admission, were collected and included in the analysis. Visual acuity was analyzed and primarily reported in Logarithm of the Minimum Angle of Resolution (logMAR) units, as this format provides a continuous and statistically appropriate scale for quantitative comparisons. Snellen decimal values were also presented for clinical interpretability.

Collected data from the Eye bank regarding the donated corneas included donor age and sex. Patients were divided into five groups based on the time interval between surgery and follow-up examination, during which postoperative data were collected: 3–6 months, 8–9 months, 10–11 months, 12–15 months, and more than 2 years. These data included postoperative BCVA and characteristics of the transplanted cornea, measured using AS-OCT (Optovue Inc., Fremont, CA, USA) and a Pentacam device (OCULUS Optikgeräte GmbH, Wetzlar, Germany).

AS-OCT provided information on the total corneal thickness and graft thickness. Pentacam measurements included the total corneal thickness, corneal densitometry (CD), and higher-order aberrations (HOAs).

CD was automatically analyzed for the entire cornea, as well as separately in the anterior (120 µm), central, and posterior (60 µm) layers, within four concentric zones (0–2 mm, 2–6 mm, 6–10 mm, and 10–12 mm). A separate value was calculated for each subgroup, resulting in a total of 20 corneal densitometry parameters. The software automatically measures the CD, which is expressed in a grayscale unit (GSU) that ranges from 0 (minimum light backscattering) to 100 (maximum light backscattering) [20]. The root-mean-square (RMS) of the corneal higher-order aberrations was assessed with Zernike analysis for the anterior corneal surface, the posterior corneal surface, and total corneal HOAs.

Categorical data were presented as absolute and relative frequencies. The normality of distribution for continuous variables was assessed using the Shapiro–Wilk test. As the continuous variables did not follow a normal distribution and due to the small sample size, nonparametric tests were used for statistical comparisons. Continuous variables were described using the median and interquartile range (IQR). Changes in visual acuity before and after surgery were analyzed using the Wilcoxon signed-rank test, with the difference and 95% confidence interval (CI) being reported. The association between visual acuity and the time elapsed from surgery to follow-up, as well as with other observed variables, was evaluated using Spearman’s rank correlation coefficient (rho). All *p* values were two-tailed, and the level of statistical significance was set at α = 0.05. Statistical analyses were performed using MedCalc^®^ Statistical Software, version 23.1.7 (MedCalc Software Ltd., Ostend, Belgium; https://www.medcalc.org; 2025). The study report was prepared in accordance with the current guidelines for reporting research in biomedicine and health sciences [21,22].

## 3. Results

The analysis included 17 eyes from 13 patients who underwent DSAEK. Of the included eyes, 6 (35.3%) were from male patients and 11 (64.7%) from female patients. The median age of treated patients (recipients) was 66 years, ranging from 58 to 77 years. Surgery was performed on the right eye in 9 cases and on the left eye in 8 cases. In 10 cases, the indication for surgery was FECD, and in 7 cases, it was PBK. Regarding the type of surgery, 10 eyes underwent isolated DSAEK, while 7 eyes underwent the triple DSAEK procedure. The median follow-up period from surgery to postoperative examination was 11 months, with a range from 3 to 32 months. Donor corneas were obtained from both male and female donors in equal proportion. The median age of donors was 64 years, with a range from 46 to 86 years (Table 1).

The postoperative visual acuity significantly improved compared to preoperative values, both in Snellen decimal units (median 0.50 vs. 0.10) and in logMAR values (median 0.30 vs. 1.00), with a statistically significant difference (*p* < 0.001 for both). The median change in Snellen visual acuity was 0.33 (95% CI: 0.25 to 0.43), while the change in logMAR was −0.64 (95% CI: −0.90 to −0.44) (Table 2). Based on the postoperative Snellen visual acuity measurements in the 17 eyes, 3 eyes (17.6%) achieved a VA of ≥0.8, while the VA of 9 eyes (52.9%) ≥0.5. The corresponding median postoperative VA was 0.50 Snellen (IQR 0.30–0.63), which equals approximately 0.30 logMAR (IQR ~0.20–0.52). For reference, the individual Snellen values ranged from 0.2 to 1.0, corresponding to logMAR values from 0.00 to 0.70.

No statistically significant differences in postoperative visual acuity were observed across different follow-up time groups (*p* = 0.22) (Table 3).

The Spearman’s rank correlation coefficient (Rho) was used to evaluate associations between postoperative visual acuity (logMAR), time since surgery, and other variables [22]. No significant correlations were found between follow-up time and observed values.

**Figure 1:**
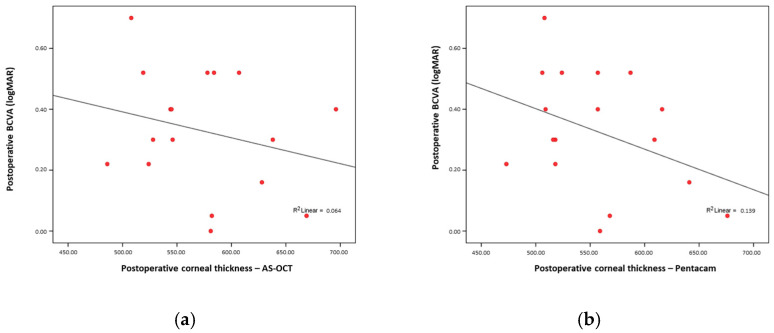
(**a**) No significant correlation between AS-OCT; (**b**) no significant correlation between postoperative corneal thickness and postoperative BCVA.

There was no significant correlation between the postoperative visual acuity and total corneal thickness, either when measured by AS-OCT (Rho = −0.227; *p* = 0.38) (Figure 1a) or by Pentacam (Rho = −0.369; *p* = 0.14) (Figure 1b).

**Figure 2:**
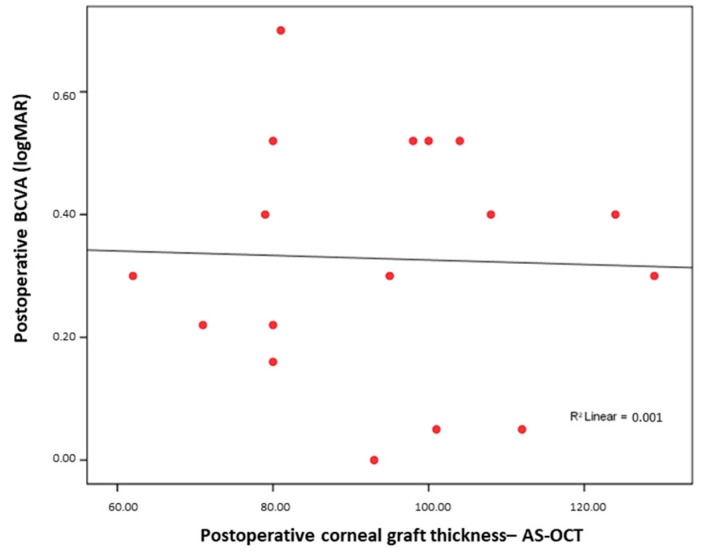
No significant correlation between AS-OCT postoperative corneal graft thickness and postoperative BCVA.

Similarly, regarding the Pentacam postoperative corneal thickness and postoperative BCVA, no significant association was found between the postoperative graft thickness and visual acuity (Rho = 0.051; *p* = 0.85) (Figure 2).

The median postoperative graft thickness measured by AS-OCT was 95 µm (IQR 80–106 µm). The total corneal thickness measured by AS-OCT was 578 µm (IQR 526–617.5 µm), and by Pentacam, it was 556 µm (IQR 512–596 µm) (Table 4).

However, several factors showed significant correlations with postoperative visual acuity.

Better postoperative visual acuity (lower logMAR values) was associated with a younger recipient age (Rho = 0.654; *p* < 0.001).

**Figure 3:**
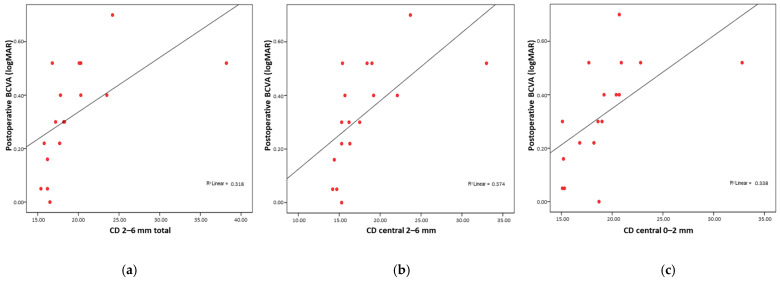
Strong significant correlation between postoperative BCVA and postoperative CD (**a**) CD 2–6 mm, (**b**) CD central 2–6 mm, (**c**) CD central 0–2 mm.

Postoperative visual acuity was worse when higher values were observed in CD total, CD central total, CD posterior total, CD 0–2 mm total, CD 2–6 mm total, CD 6–10 mm total, CD anterior 2–6 mm, CD anterior 6–10 mm, CD central 0–2 mm, CD central 2–6 mm, CD central 6–10 mm, CD posterior 0–2 mm, CD posterior 2–6 mm, and CD posterior 6–10 mm. A stronger correlation with visual acuity was found for CD 2–6 mm total (Rho = 0.795; *p* < 0.001) (Figure 3a), CD central 2–6 mm (Rho = 0.791; *p* < 0.001) (Figure 3b), and CD central 0–2 mm (Rho = 0.730; *p* < 0.001) (Figure 3c).

**Figure 4:**
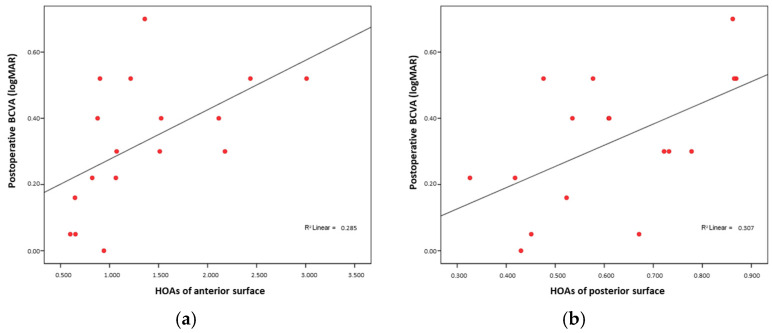
(**a**) BCVA and anterior HOA correlation; (**b**) BCVA and posterior HOA correlation.

Additionally, higher anterior and posterior higher-order aberrations (HOA anterior and HOA posterior) were also associated with poorer visual outcomes (Rho = 0.624, *p* = 0.01; and Rho = 0.556, *p* = 0.02, respectively) (Figure 4a,b).

In this case, since lower logMAR values represent better visual acuity, a positive Spearman’s Rho indicates a direct proportional relationship—as logMAR increases (worse visual acuity), the other variable also increases. A negative Rho, on the other hand, signifies an inverse relationship, meaning that as logMAR increases, the other variable decreases. All statistical tests were two-tailed, with a significance level set at α = 0.05. Correlation of postoperative visual acuity (logMAR) and time since surgery with all of the examined parameters are shown in Table 5. 

## 4. Discussion

This study aimed to examine the relationship between postoperative visual acuity following DSAEK surgery and various morphological and optical parameters, measured postoperatively.

The results demonstrated that higher values of CD, especially in the central and posterior cornea, as well as higher values of anterior and posterior surface HOAs, were significantly associated with worse visual outcomes. In contrast, traditionally monitored variables such as the corneal and graft thickness did not show a statistically significant correlation with visual acuity.

Our findings support existing research suggesting that the graft or total corneal thickness alone has limited predictive value for postoperative visual acuity, particularly in cases where corneal clarity is restored but visual function remains suboptimal. No significant correlation was found between the graft thickness, measured postoperatively by AS-OCT, and visual outcomes. Although some studies align with these results [15,16], others argue that thinner grafts, such as those used in UT-DSAEK, may contribute to improved visual performance [23,24].

Better postoperative visual acuity was significantly associated with a younger recipient age, a finding that is consistent with previous studies in the literature that have reported age as an important factor influencing visual recovery after EK [23].

No significant difference in postoperative visual acuity was observed between eyes that underwent DSAEK alone and those that received the triple DSAEK procedure, nor between eyes that were operated for FECD and those with PBK. These findings are consistent with the existing literature and may suggest that postoperative visual outcomes are not significantly influenced by IOL implantation or the underlying indication for surgery [25,26].

This study’s detailed analysis of CD, which separately evaluated the relationship between postoperative visual acuity and CD values measured in distinct corneal layers (anterior, central, and posterior) and across specific concentric zones, resulted in a total of 20 individual CD measurements. This comprehensive approach provides a more nuanced understanding of how localized changes in corneal transparency may influence visual outcomes following DSAEK surgery.

Changes in corneal transparency may influence visual outcomes following DSAEK surgery. Our findings suggest that, beyond total CD, the strongest association with postoperative visual acuity is found in CD values that are measured in the central and posterior layers of the cornea and in the central concentric zones, as opposed to the peripheral regions.

This may be explained by the fact that the visual axis passes through the central part of the cornea, making this region more critical for image formation on the retina and therefore more influential on visual acuity. Additionally, the underlying conditions that indicated surgery, primarily endothelial pathologies, affect the deeper, posterior layers of the cornea, which further supports the observed association between changes in central and posterior corneal transparency and postoperative visual outcomes.

In the literature, considerable attention has been given to the role of interface haze, formed at the junction between the donor and recipient cornea, which may help explain the strong influence of the central layer CD on visual acuity observed in this study, particularly if the central layer measurements encompassed the graft–host interface in cases where the graft thickness exceeded 60 µm. Some studies have also considered the possibility that, due to gradual deturgescence of DSAEK grafts over time, densitometry measurements of the posterior 60 µm layer may capture the stromal–stromal interface. This could provide valuable insight into differences in graft–host interface clarity, particularly when comparing DMEK and UT-DSAEK procedures [18]. Other studies have specifically attempted to measure the densitometry of this interface haze [10,27]. However, a key limitation of such research, as well as of the Pentacam system itself, is the lack of an automated method for isolating and measuring CD in the interface region. Instead, manual adjustment of the measurement zone is required, which reduces both precision and reproducibility, thereby limiting the reliability of these assessments.

This study also demonstrated a statistically significant correlation between both anterior and posterior surface HOAs and postoperative visual acuity, which is consistent with findings reported in the literature [16,28,29]. However, no significant correlation was observed between total HOAs and visual outcomes, which may be attributed to the limited sample size.

A relevant limitation of this study is the small sample size, which may reduce the statistical power and limit the generalizability of the findings. Future studies with larger cohorts are necessary to validate and expand upon these results.

One more notable limitation of this study is the absence of preoperative measurements of corneal densitometry (CD) and higher-order aberrations (HOAs). However, this limitation is partly methodological, as most patients included in the study presented with advanced corneal edema, bullous changes, central guttata, and Descemet’s membrane folds prior to surgery. These pathological features are known to significantly distort imaging results obtained via Scheimpflug-based devices such as the Pentacam, making reliable preoperative assessment of CD and HOAs technically challenging and clinically inconsistent. Consequently, comparisons with baseline values may not have provided meaningful or reproducible insights in this patient population [25]. Nonetheless, future studies would benefit from incorporating more comprehensive preoperative diagnostic assessments, where possible, as this could provide valuable baseline data for understanding individual variations in surgical outcomes and the progression of corneal changes.

From a clinical perspective, the inclusion of CD and HOA measurements in postoperative evaluation protocols may provide added value beyond standard structural assessments. Identifying increased CD or elevated HOAs—particularly in the central cornea—can help explain suboptimal visual outcomes, even when graft attachment and clarity appear normal on slit-lamp examination. This may guide clinicians in setting more realistic expectations for patients during postoperative counseling. Moreover, longitudinal monitoring of CD and HOAs may serve as a useful tool to detect subtle changes in graft–host interface quality or early signs of graft failure, thereby informing tailored follow-up intervals and possible interventions.

## 5. Conclusions

This study highlights the clinical significance of both corneal densitometry and high-order aberrations as predictors of postoperative visual acuity in patients undergoing DSAEK surgery. The results indicate that these corneal imaging modalities are a more significant factor for postoperative visual acuity than commonly measured parameters, such as graft thickness or overall corneal thickness. These findings highlight the importance of incorporating advanced optical assessments into postoperative evaluation protocols. Further research should include a larger patient sample and additional parameters, which could further confirm the significance of these findings and contribute to a better understanding of factors influencing postoperative visual outcomes.

## Figures and Tables

**Table 1:** General and clinical characteristics.

Variable	Value
Male recipient [n (%)]	6/17 (35%)
Female recipient [n (%)]	11/17 (65%)
Male donor [n (%)]	8/17 (47%)
Female donor [n (%)]	9/17 (53%)
Recipient age [years, median (IQR)]	66 (62–74)
Donor age [years, median (IQR)]	64 (58–69)
Right operated eye [n (%)]	9/17 (53%)
Left operated eye [n (%)]	8/17 (47%)
Time from surgery to follow-up [months, median (IQR)]	11 (9–15)
Follow-up 3–6 months after surgery [n (%)]	3/17 (18%)
Follow-up 8–9 months aftersurgery [n (%)]	3/17 (18%)
Follow-up 10–11 months after surgery [n (%)]	3/17 (18%)
Follow-up 12–15 months after surgery [n (%)]	5/17 (28%)
Follow-up > 2 years after surgery [n (%)]	3/17 (18%)
FECD [n (%)]	10/17 (59%)
PBK [n (%)]	7/17 (41%)
DSAEK [n (%)]	10/17 (59%)
Triple DSAEK [n (%)]	7/17 (41%)

**Table 2:** Differences in visual acuity before and after surgery.

Visual Acuity Measure	Preoperative Median (IQR)	Postoperative Median (IQR)	Difference	95% CI	*p* *
Snellen	0.10 (0.04–0.23)	0.50 (0.30–0.63)	0.33	0.25 to 0.43	<0.001
logMAR	1.0 (0.66–1.4)	0.30 (0.21–0.52)	−0.64	−0.90 to −0.44	<0.001

* Wilcoxon test.

**Table 3:** Differences in visual acuity according to time since surgery.

Median (IQR) by Follow-Up Time	3–6 Months	8–9 Months	10–11 Months	12–15 Months	>2 Years	*p* Value *
Postoperative visual acuity (Snellen)	0.40 (0.33–0.48)	0.30 (0.23–0.53)	0.90 (0.60–0.98)	0.40 (0.30–0.63)	0.50 (0.43–0.80)	0.22
Postoperative visual acuity (logMAR)	0.40 (0.33–0.49)	0.52 (0.30–0.66)	0.05 (0.01–0.24)	0.40 (0.20–0.52)	0.30 (0.11–0.38)	0.22

* Kruskal–Wallis test.

**Table 4:** Postoperative corneal thickness measurements.

Parameter	Median (Interquartile Range)	Minimum–Maximum
Total corneal thickness on AS-OCT [µm]—postoperative	578 (526–617.5)	486–696
Total corneal thickness on Pentacam [µm]—postoperative	557 (512.5–598)	473–676
Graft thickness on AS-OCT [µm]—postoperative	95 (80–106)	62–129

**Table 5:** Correlation of postoperative visual acuity (logMAR) and time since surgery with examined parameters (statistically significant correlations (*p* < 0.05) are shown in bold).

Parameter	logMAR BCVA (Rho, *p*)	Time Since Surgery (Rho, *p*)
Recipient age	0.654 (<0.001)	−0.417 (0.10)
Donor age	0.060 (0.82)	−0.246 (0.34)
Postoperative graft thickness (AS-OCT)	0.051 (0.85)	0.188 (0.47)
Postoperative total corneal thickness (AS-OCT)	−0.227 (0.38)	0.156 (0.55)
Postoperative total corneal thickness (Pentacam)	−0.369 (0.14)	0.085 (0.75)
CD total	0.551 (0.02)	−0.119 (0.65)
CD anterior total	0.418 (0.10)	0.039 (0.88)
CD central total	0.556 (0.02)	−0.208 (0.42)
CD posterior total	0.584 (0.01)	−0.132 (0.61)
CD 0–2 mm total	0.660 (<0.001)	−0.072 (0.78)
CD 2–6 mm total	0.795 (<0.001)	−0.192 (0.46)
CD 6–10 mm total	0.649 (<0.001)	−0.215 (0.41)
CD 10–12 mm total	−0.117 (0.65)	−0.072 (0.78)
CD anterior 0–2 mm	0.333 (0.19)	0.474 (0.05)
CD anterior 2–6 mm	0.590 (0.01)	0.301 (0.24)
CD anterior 6–10 mm	0.677 (<0.001)	−0.215 (0.41)
CD anterior 10–12 mm	−0.148 (0.57)	−0.036 (0.89)
CD central 0–2 mm	0.730 (<0.001)	−0.024 (0.93)
CD central 2–6 mm	0.791 (<0.001)	−0.286 (0.27)
CD central 6–10 mm	0.654 (<0.001)	−0.289 (0.26)
CD central 10–12 mm	−0.027 (0.92)	−0.162 (0.53)
CD posterior 0–2 mm	0.644 (0.01)	−0.256 (0.32)
CD posterior 2–6 mm	0.613 (0.01)	−0.332 (0.19)
CD posterior 6–10 mm	0.590 (0.01)	0.027 (0.92)
CD posterior 10–12 mm	−0.065 (0.80)	0.002 (0.99)
HOA total	0.171 (0.51)	0.073 (0.78)
HOA anterior	0.624 (0.01)	−0.043 (0.87)
HOA posterior	0.556 (0.02)	0.064 (0.81)

## Data Availability

The data supporting the findings of this study are not publicly available due to the presence of personal information and ethical considerations.

## Abbreviations

The following abbreviations are used in this manuscript:

EK	Endothelial Keratoplasty
FECD	Fuchs Endothelial Corneal Dystrophy
BK	Bullous Keratopathy
PKP	Penetrating Keratoplasty
DSAEK	Descemet Stripping Automated Endothelial Keratoplasty
IOL	Intraocular Lens
HOAs	Higher-Order Aberrations
DMEK	Descemet’s Membrane Endothelial Keratoplasty
UT-DSAEK	Ultrathin Descemet Stripping Automated Endothelial Keratoplasty
CD	Corneal Densitometry
AS-OCT	Anterior Segment Optical Coherence Tomography
BCVA	Best-Corrected Visual Acuity
PBK	Pseudophakic Bullous Keratopathy
GSU	Grayscale Unit
RMS	Root-Mean-Square
logMAR	Logarithm of the Minimum Angle of Resolution
CI	Confidence Interval
Rho	Spearman’s Rank Correlation Coefficient
